# Cortical entrainment to continuous speech: functional roles and interpretations

**DOI:** 10.3389/fnhum.2014.00311

**Published:** 2014-05-28

**Authors:** Nai Ding, Jonathan Z. Simon

**Affiliations:** ^1^Department of Psychology, New York UniversityNew York, NY, USA; ^2^Department of Electrical and Computer Engineering, University of Maryland College Park, College ParkMD, USA; ^3^Department of Biology, University of Maryland College Park, College ParkMD, USA; ^4^Institute for Systems Research, University of Maryland College Park, College ParkMD, USA

**Keywords:** auditory cortex, entrainment of rhythms, speech intelligibility, speech perception in noise, speech envelope, cocktail party problem

## Abstract

Auditory cortical activity is entrained to the temporal envelope of speech, which corresponds to the syllabic rhythm of speech. Such entrained cortical activity can be measured from subjects naturally listening to sentences or spoken passages, providing a reliable neural marker of online speech processing. A central question still remains to be answered about whether cortical entrained activity is more closely related to speech perception or non-speech-specific auditory encoding. Here, we review a few hypotheses about the functional roles of cortical entrainment to speech, e.g., encoding acoustic features, parsing syllabic boundaries, and selecting sensory information in complex listening environments. It is likely that speech entrainment is not a homogeneous response and these hypotheses apply separately for speech entrainment generated from different neural sources. The relationship between entrained activity and speech intelligibility is also discussed. A tentative conclusion is that theta-band entrainment (4–8 Hz) encodes speech features critical for intelligibility while delta-band entrainment (1–4 Hz) is related to the perceived, non-speech-specific acoustic rhythm. To further understand the functional properties of speech entrainment, a splitter’s approach will be needed to investigate (1) not just the temporal envelope but what specific acoustic features are encoded and (2) not just speech intelligibility but what specific psycholinguistic processes are encoded by entrained cortical activity. Similarly, the anatomical and spectro-temporal details of entrained activity need to be taken into account when investigating its functional properties.

## INTRODUCTION

**FIGURE 1:**
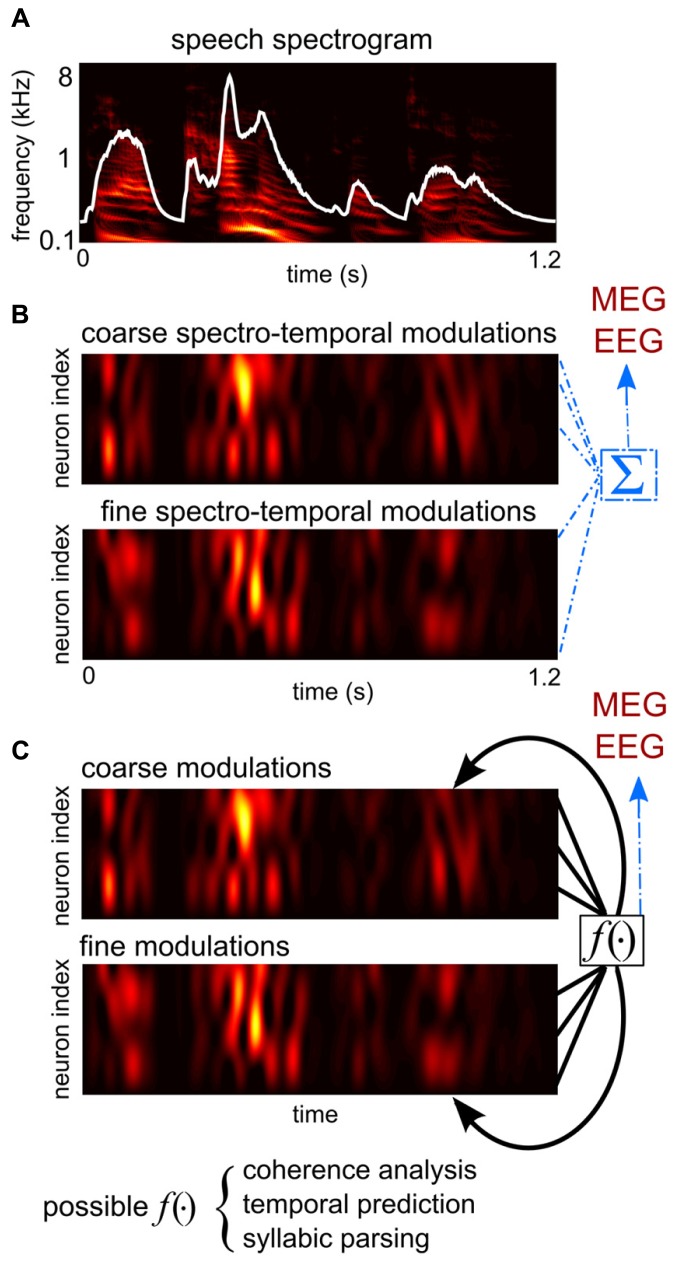
**A schematic illustration of hypotheses proposed to explain the generation of cortical entrainment to the speech envelope. (A)** The spectro-temporal representation of speech, obtained from a cochlear model (Yang et al., 1992). The broad-band temporal envelope of speech, the sum of the spectro-temporal representation over frequency, is superimposed in white. **(B)** An illustration of the collective feature tracking hypothesis and the onset tracking hypothesis. The colored images show time courses of the dendritic activity of two example groups of neurons, hypothetically in primary and associative auditory areas. One group encodes the slow temporal modulations and coarse spectral modulations of sound intensity, i.e., the spectro-temporal envelope of speech, which contain major phonetic cues. The other group encodes the slow temporal changes of cues computed from the spectro-temporal fine structure, e.g., the pitch contour and the trajectory of the sound source location. According to the collective feature tracking hypothesis, magnetoencephalography (MEG)/electroencephalography (EEG) measurements are the direct sum of dendritic activity across all such neural populations in primary and associative auditory areas. The onset tracking hypothesis is similar, but instead neurons encoding the temporal edges of speech dominate cortical activity and thus drive MEG/EEG measurable responses. **(C)** An illustration of the syllabic parsing hypothesis and the sensory selection hypotheses. These hypotheses assume certain computations that integrate over distributively-represented auditory features. The syllable parsing hypothesis hypothesizes neural operations integrating features belonging to the same syllable. The sensory selection hypotheses propose either a temporal coherence analysis or a temporal predictive analysis.

Speech recognition is a process that maps an acoustic signal onto the underlying linguistic meaning. The acoustic properties of speech are complex and contain temporal dynamics on several time scales (Rosen, 1992; Chi et al., 2005). The time scale most critical for speech recognition is on the order of hundreds of milliseconds (1–10 Hz), and the temporal fluctuations on this time scale are usually called the *temporal envelope* (**Figure 1A**). Single neuron neurophysiology from animal models has shown that neurons in primary auditory cortex encode the analogous temporal envelope of other non-speech sounds by phase locked neural firing (Wang et al., 2003). In contrast, the finer scale acoustic properties that decide the pitch and timbre of speech at each time moment (acoustic fragments lasting a few 100 ms) are likely to be encoded using a spatial code, by either individual neurons (Bendor and Wang, 2005) or spatial patterns of cortical activity (Walker et al., 2011).

In the last decade or so, cortical entrainment to the temporal envelope of speech has been demonstrated in humans using magnetoencephalography (MEG; Ahissar et al., 2001; Luo and Poeppel, 2007), electroencephalography (EEG; Aiken and Picton, 2008), and electrocorticography (ECoG; Nourski et al., 2009). This envelope following response can be recorded from subjects listening to sentences or spoken passages and therefore provides an online marker of neural processing of continuous speech. Envelope entrainment has mainly been seen in the waveform of low-frequency neural activity (<8 Hz) and in the power envelope of high-gamma activity (Pasley et al., 2012; Zion Golumbic et al., 2013). Although the phenomenon of envelope entrainment has been well established, its underlying neural mechanisms, and functional roles remain controversial. It is still under debate whether entrained cortical activity is more closely tied to the physical properties of the acoustic stimulus or to higher level language related processing that is directly related to speech perception. A number of studies have shown that cortical entrainment to speech is strongly modulated by top–down cognitive functions such as attention (Kerlin et al., 2010; Ding and Simon, 2012a; Mesgarani and Chang, 2012; Zion Golumbic et al., 2013) and therefore is not purely a bottom-up response. On the other hand, cortical entrainment to the sound envelope is seen for non-speech sound (Lalor et al., 2009; Hämäläinen et al., 2012; Millman et al., 2012; Wang et al., 2012; Steinschneider et al., 2013) and therefore does not rely on speech-specific neural processing. In this article, we first summarize a number of hypotheses about the functional roles of envelope entrainment, and then review the literature about how envelope entrainment is affected by speech intelligibility.

## FUNCTIONAL ROLES OF CORTICAL ENTRAINMENT

A number of hypotheses have been proposed about what aspects of speech, ranging from its acoustic features to its linguistic meaning, are encoded by entrained cortical activity. A few dominant hypotheses are summarized and compared (**Table 1**). Other unresolved questions about cortical neural entrainment, e.g., what the biophysical mechanisms generating cortical entrainment are, and whether entrained neural activity is related to spontaneous neural oscillations, are not covered here (see discussions in e.g., Schroeder and Lakatos, 2009; Howard and Poeppel, 2012; Ding and Simon, 2013b).

**Table 1:** A summary of major hypotheses about the functional roles of cortical entrainment to speech.

Hypothesis	Underlying neural computations	Reference
Onset tracking	Temporal edge detection	Howard and Poeppel (2010)
Collective feature tracking	Spectro-temporal feature coding	Ding and Simon (2012b), Ghitza et al. (2012)
Syllabic parsing	Binding features of the same syllable; discretization	Giraud and Poeppel (2012), Ghitza (2013)
Sensory selection I	Temporal coherence-based binding of auditory features	Shamma et al. (2011), Ding and Simon (2012a)
Sensory selection II	Modulation of neuronal excitability; temporal prediction	Schroeder and Lakatos (2009)

### ONSET TRACKING HYPOTHESIS

Speech is dynamic and is full of acoustic “edges,” e.g., onsets and offsets. These edges usually occur at syllable boundaries and are well characterized by the speech envelope. It is well known that a reliable macroscopic brain response can be evoked by an acoustic edge. Therefore, it has been proposed that neural entrainment to the speech envelope is a superposition of discrete, edge/onset related brain responses (Howard and Poeppel, 2010). Consistent with this hypothesis, it has been shown that the sharpness of acoustic edges, i.e., how quickly sound intensity increases, strongly influences cortical tracking of the sound envelope (Prendergast et al., 2010; Doelling et al., 2014). A challenge of this hypothesis, however, is that speech is continuously changing and it remains a problem as to which acoustic transients can be counted as edges.

If this hypothesis is true, a question naturally follows about whether envelope entrainment can provide insights that cannot be learned using the traditional event-related response approach. The answer is yes. Cortical responses, including edge/onset related auditory evoked responses, are stimulus-dependent, and quickly adapt to the spectro-temporal structure of the stimulus (Zacharias et al., 2012; Herrmann et al., 2014). Therefore, even if envelope entrainment is just a superposition of event-related responses, it can still provide insights about the properties of cortical activity when it is adapted to the acoustic properties of speech.

### COLLECTIVE FEATURE TRACKING HYPOTHESIS

When sound enters the ear, it is decomposed into narrow frequency bands in the auditory periphery and is further decomposed into multi-scale acoustic features in the central auditory system, such as pitch, sound source location information, and coarse spectro-temporal modulations (Shamma, 2001; Ghitza et al., 2012). In speech, most acoustic features coherently fluctuate in time and these coherent fluctuations are captured by the speech envelope. If a neuron or a neural population encodes an acoustic feature, its activity is synchronized to the strength of that acoustic feature. As a result, neurons or neural networks that are tuned to coherently fluctuating speech features are activated coherently (Shamma et al., 2011).

Analogously to the speech envelope being the summation of the power of all speech features at each time moment, the large-scale neural entrainment to speech measured by MEG/EEG can be the summation of neural activity tracking different acoustic features of speech (**Figure 1B**). It is therefore plausible to hypothesize that macroscopic speech entrainment is a passive summation of microscopic neural tracking of acoustic features across neurons/networks (Ding and Simon, 2012b). Based on this hypothesis, the MEG/EEG speech entrainment is a marker of a collective cortical representation of speech but does not play any additional roles in regulating neuronal activity.

The onset tracking hypothesis can be viewed as a special case of the collective feature tracking hypothesis, when the acoustic features driving cortical responses are restricted to a set of discrete edges. The collective feature tracking hypothesis, however, is more general since it allows features to be continuously changing and also incorporates features that are not associated with sharp intensity changes, such as changes in the pitch contour (Obleser et al., 2012), and sound source location. Under the onset tracking hypothesis, entrained neural activity is a superposition of onset/edge-related auditory evoked responses. Under the more general collective feature tracking hypothesis, at a first-order approximation, entrained activity is a convolution between speech features, e.g., the temporal envelopes in different narrow frequency bands, and the corresponding response functions, e.g., the response evoked by a very brief tone pip in the corresponding frequency band (Lalor et al., 2009; Ding and Simon, 2012b).

### SYLLABIC PARSING HYPOTHESIS

During speech recognition, the listener must segment a continuous acoustic signal into a sequence of discrete linguistic symbols, into the units of, e.g., phonemes, syllables or words. The boundaries between phonemes, and especially syllables, are relatively well encoded by the speech envelope (Stevens, 2002; Ghitza, 2013, see also Cummins, 2012). Furthermore, the average syllabic rate ranges between 5 and 8 Hz across languages (Pellegrino et al., 2011) and the rate for stressed syllables is below 4 Hz for English (Greenberg et al., 2003). Therefore it has been hypothesized that neural entrainment to the speech envelope plays a role in creating a syllabic level, discrete, representation of speech (Giraud and Poeppel, 2012). In particular, it has been hypothesized that each cycle of the cortical theta oscillation (4–8 Hz) is aligned to the portion of speech signal in between of two vowels, corresponding to two adjacent peaks in the speech envelope. Auditory features within a cycle of theta oscillation are then used to decode the phonetic information of speech (Ghitza, 2011, 2013). Therefore, according to this hypothesis, speech entrainment does not only passively track acoustic features but also reflects the language-based packaging of speech into syllable size chunks. Since syllables play different roles in segmenting syllable-timed language and stress-timed language (Cutler et al., 1986), further cross-language research may further elucidate which of these neural processes are represented in envelope tracking activity.

### SENSORY SELECTION HYPOTHESIS

In everyday listening environments, speech is often embedded in a complex acoustic background. Therefore, to understand speech, a listener must segregate speech from the listening background and process it selectively. A useful strategy for the brain would be to find and selectively process moments in time (or spectro-temporal instances in a more general framework) that are dominated by speech and ignore the moments dominated by the background (Wang, 2005; Cooke, 2006). In other words, the brain might robustly encode speech by taking glimpses at the temporal (or spectro-temporal) features that contain critical speech information. The rhythmicity of speech (Schroeder and Lakatos, 2009; Giraud and Poeppel, 2012), and the temporal coherence between acoustic features (Shamma et al., 2011), are both reflected by the speech envelope and so become critical cues for the brain to decide where the useful speech information lies. Therefore, envelope entrainment may play a critical role in the neural segregation of speech and the listening background.

In a complex listening environment, cortical entrainment to speech has been found to be largely invariant to the listening background (Ding and Simon, 2012a; Ding and Simon, 2013a). Two possible functional roles have been hypothesized for the observed background-invariant envelope entrainment. One is that the brain uses temporal coherence to bind together acoustic features belonging to the same speech stream and envelope entrainment may reflect computations related to this coherence analysis (Shamma et al., 2011; Ding and Simon, 2012a). The other is that envelope entrainment is used by the brain to predict which moments contain more information about speech than the acoustic background and then guide the brain to selectively process those moments (Schroeder et al., 2008; Schroeder and Lakatos, 2009; Zion Golumbic et al., 2012).

### WHICH HYPOTHESIS IS TRUE? AN ANALYSIS-BY-SYNTHESIS ACCOUNT OF SPEECH PROCESSING

Speech processing is a complicated process that can be roughly divided into an analysis stage and a synthesis stage. In the analysis stage, speech sounds are decomposed into primitive auditory features, a process that starts from the cochlea and applies mostly equally to the auditory encoding of both speech and non-speech sounds. A later synthesis stage, in contrast, combines multiple auditory features to create speech perception, including, e.g., binding spectro-temporal cues to determine phonemic categories, or integrating multiple acoustic cues to segregate a target speech stream from an acoustic background. The onset tracking hypothesis and the collective feature tracking hypothesis both view speech entrainment as a passive auditory encoding mechanism belonging to the analysis stage. Note, however, that the analysis stage does include some integration over separately represented features also. For example, neural processing of pitch and spectral modulations requires integrating information across frequency. Functionally, however, the purpose of integrating features in the analysis stage is to extract higher level auditory features rather than to construct linguistic/perceptual entities.

The syllabic parsing hypothesis and the sensory selection hypothesis propose functional roles of cortical entrainment in the synthesis stage. They hypothesize that cortical entrainment is involved in combining features into linguistic units, e.g., syllables, or perceptual units, e.g., speech streams (**Figure 1C**). These additional functional roles may be implemented in two ways: an active mechanism would be one that entrained cortical activity, as a large-scale voltage fluctuation, directly regulating syllabic parsing or sensory selection (Schroeder et al., 2008; Schroeder and Lakatos, 2009). A passive mechanism would be one where neural computations related to syllabic parsing or sensory selection would generate spatially coherent neural signals that are measurable by macroscopic recording tools.

Although clearly distinctive from each other, the four hypotheses may all be true for different functional areas of the brain and describe different neural generators for speech entrainment. Onset detection, feature tracking, syllabic parsing, and sensory selection are all neural computations necessary for speech recognition and all of them are likely to be synchronized to the speech rhythm carried by the envelope. Therefore, these neural computations may all be reflected by cortical entrainment to speech, and may only differ in their fine-scale neural generators. It remains unclear, however, whether these fine-scale neural generators can be resolved by macroscopic recording tools such as MEG and EEG.

Future studies are needed to explicitly test these hypotheses, or explicitly modify them, to determine which specific acoustic features and which specific psycholinguistic processes are relevant to cortical entrainment. For example, to dissociate the onset tracking hypothesis and the collective feature tracking hypothesis, one approach is to create explicit computational models for them and test which model would fit the data better. To test the syllabic parsing hypothesis, it will be important to calculate the correlation between cortical entrainment and relevant behavioral measures, e.g., misallocation of syllable boundaries (Woodfield and Akeroyd, 2010). To test the sensory selection hypothesis, stimuli that vary in their temporal probability or coherence among spectro-temporal features are likely to be revealing.

## ENVELOPE ENTRAINMENT AND SPEECH INTELLIGIBILITY

### ENTRAINMENT AND ACOUSTIC MANIPULATION OF SPEECH

As indicated by its name, envelope entrainment is correlated with the speech envelope, an acoustic property of speech. Nevertheless, neural encoding of speech must underlie the ultimate goal of decoding its meaning. Therefore, it is critical to identify if cortical entrainment to speech is related to any behavioral measure during speech recognition, such as speech intelligibility.

A number of studies have compared cortical activity entrained to intelligible speech and unintelligible speech. One approach is to vary the acoustic stimulus and analyze how cortical entrainment changes within individual subjects. Some studies have found that cortical entrainment to normal sentences is similar to cortical entrainment to sentences that are played backward in time (Howard and Poeppel, 2010; Peña and Melloni, 2012; though see Gross et al., 2013).

A second way to reduce intelligibility is to introduce different types of acoustic interference. When speech is presented together with stationary noise, delta-band (1–4 Hz) cortical entrainment to the speech is found to be robust to noise until the listeners can barely hear speech, while theta-band (4–8 Hz) entrainment decreases gradually as the noise level increases (Ding and Simon, 2013a). In this way, theta-band entrainment is correlated with noise level and also speech intelligibility, but delta-band entrainment is not. When speech is presented together with a competing speech stream, cortical entrainment is found to be robust against the level of the competing speech stream even though intelligibility drops (Ding and Simon, 2012a; theta- and delta-band activity was not analyzed separately there).

A third way to reduce speech intelligibility is to degrade the spectral resolution through noise-vocoding, which destroys spectro-temporal fine structure but preserves the temporal envelope (Shannon et al., 1995). When the spectral resolution of speech decreases, it has been shown that theta-band cortical entrainment reduces (Peelle et al., 2013; Ding et al., 2014) but delta-band entrainment enhances (Ding et al., 2014). In contrast, when background noise is added to speech and the speech-noise mixture is noise vocoded, it is found that both delta- and theta-band entrainment is reduced by vocoding (Ding et al., 2014).

A fourth way to vary speech intelligibility is to directly manipulate the temporal envelope (Doelling et al., 2014). When the temporal envelope in the delta-theta frequency range is corrupted, cortical entrainment in the corresponding frequency bands degrades and so does speech intelligibility. When a processed speech envelope is used to modulate a broadband noise carrier, the stimulus is not intelligible but reliable cortical entrainment is nevertheless seen.

In many of these studies investigating the correlation between cortical entrainment and intelligibility, a common issue is that stimuli which differ in intelligibly also differ in acoustic properties. This makes it is difficult to determine if changes in cortical entrainment arise from changes in speech intelligibility or from changes in acoustic properties. For example, speech syllables generally have a sharper onset than offset, so reversing speech in time changes those temporal characteristics. Similarly, when the spectral resolution is reduced, neurons tuned to fine spectral features are likely to be deactivated. Therefore, based on the studies reviewed here, it can only be tentatively concluded that, when critical speech features are manipulated, speech intelligibility, and theta-band entrainment are affected in similar ways while delta-band entrainment is not. It remains unclear about whether speech intelligibility causally modulates cortical entrainment or that auditory encoding, reflected by cortical entrainment, influences downstream language processing and therefore become indirectly related to intelligibility.

### VARIABILITY BETWEEN LISTENERS

A second approach to address the correlation between neural entrainment and speech intelligibility is to investigate the variability across listeners. Peña and Melloni (2012) compared neural responses in listeners who speak the tested language and listeners who do not speak the tested language. It was found that language understanding does not significantly change the low-frequency neural responses, but it does change high-gamma band neural activity. Within the group of native speakers, the intelligibility score still varied broadly in the challenging listening conditions. Delta-band, but not theta-band, cortical entrainment has been shown to correlate with intelligibility scores for individual listeners in a number of studies (Ding and Simon, 2013a; Ding et al., 2014; Doelling et al., 2014). The advantage of investigating inter-subject variability is that it avoids modifications of the sound stimuli. Nevertheless, it still cannot identify whether the individual differences in speech recognition arise from the individual differences in auditory processing (Ruggles et al., 2011), language related processing, or cognitive control.

The speech intelligibility approach in general, suffers from a drawback that it is the end point of the entire speech recognition chain, and is not targeted at specific linguistic computations, e.g., allocating the boundaries between syllables. Furthermore, when the acoustic properties of speech are degraded, speech recognition requires additional cognitive control and the involved neural processing networks adapt (Du et al., 2011; Wild et al., 2012; Erb et al., 2013; Lee et al., 2014). Therefore, just from a change in speech intelligibility, it is difficult to trace what kinds of neural processing are affected.

### DISTINCTIONS BETWEEN DELTA- AND THETA-BAND ENTRAINMENT

In summary of these different approaches, when the acoustic properties of speech are manipulated, theta-band entrainment often shows changes that correlate with speech intelligibility. For the same stimulus, however, the speech intelligibility measured from individual listeners is often correlated with delta-band entrainment. To explain this dichotomy, here we hypothesize that theta-band entrainment encodes syllabic-level acoustic features critical for speech recognition, while delta-band entrainment is more closely related to the perceived acoustic rhythm rather than the phonemic information of speech. This hypothesis is also consistent with the fact that speech modulations between 4 and 8 Hz are critical for intelligibility (Drullman et al., 1994a,b; Elliott and Theunissen, 2009) while temporal modulations below 4 Hz include prosodic information of speech (Goswami and Leong, 2013) and it is the frequency range important for music rhythm perception (Patel, 2008; Farbood et al., 2013).

### ENVELOPE ENTRAINMENT TO NON-SPEECH SOUNDS

Although speech envelope entrainment may show correlated changes with speech intelligibility when the acoustic properties of speech are manipulated, speech intelligibility is probably not a major driving force for envelope entrainment. A critical evidence is that envelope entrainment can be observed for non-speech sounds in humans and both speech and non-speech sounds in animals. Here, we briefly review human studies on envelope entrainment for non-speech sounds (see e.g., Steinschneider et al., 2013 for a comparison between envelope entrainment in human and animal models).

Traditionally, envelope entrainment has been studied using the auditory steady-state response (aSSR), a periodic neural response tracking the stimulus repetition rate or modulation rate. An aSSR at a given frequency can be elicited by, e.g., a click or tone-pip train repeating at the same frequency (Nourski et al., 2009; Xiang et al., 2010), and by amplitude or frequency modulation at that frequency (Picton et al., 1987; Ross et al., 2000; Wang et al., 2012). Although the cortical aSSR can be elicited in a broad frequency range (up to ~100 Hz), speech envelope entrainment is likely to be related to the slow aSSR in the corresponding frequency range, i.e., below 10 Hz (see Picton, 2007 for a review of the robust aSSR of 40 Hz and above). More recently, cortical entrainment has also been demonstrated for sounds modulated by an irregular envelope (Lalor et al., 2009). Low-frequency (<10 Hz) cortical entrainment to non-speech sound shares many properties with cortical entrainment to speech. For example, when envelope entrainment is modeled using a linear system-theoretic model, the neural response is qualitatively similar for speech (Power et al., 2012) and amplitude-modulated tones (Lalor et al., 2009). Furthermore, low-frequency (<10 Hz) cortical entrainment to non-speech sound is also strongly modulated by attention (Elhilali et al., 2009; Power et al., 2010; Xiang et al., 2010), and the phase of entrained activity is predictive of listeners’ performance in some sound-feature detection tasks (Henry and Obleser, 2012; Ng et al., 2012).

## SUMMARY

Cortical entrainment to the speech envelope provides a powerful tool to investigate online neural processing of continuous speech. It greatly extends the traditional event-related approach that can only be applied to analyze the response to isolated syllables or words. Although envelope entrainment has attracted researchers’ attention in the last decade, it is still a less well-characterized cortical response than event-related responses. The basic phenomenon of envelope entrainment has been reliably seen in EEG, MEG, and ECoG, even at the single-trial level (Ding and Simon, 2012a; O’Sullivan et al., 2014). Hypotheses have been proposed about the neural mechanisms generating cortical entrainment and its functional roles, but these hypotheses remain to be explicitly tested. To test these hypotheses, a computational modeling approach is likely to be effective. For example, rather than just calculating the correlation between neural activity and the speech envelope, more explicit computational models can be proposed and used to fit the data (e.g., Ding and Simon, 2013a). Furthermore, to understand what linguistic computations are achieved by entrained cortical activity, more fine-scaled behavioral measures are likely to be required, e.g., measures related to syllable boundary allocation rather than the general measure of intelligibility. Finally, the anatomical, temporal, and spectral specifics of cortical entrainment should be taken into account when discussing its functional roles (Peña and Melloni, 2012; Zion Golumbic et al., 2013; Ding et al., 2014).

## AUTHOR CONTRIBUTIONS

Nai Ding and Jonathan Z. Simon wrote and approved the paper.

## Conflict of Interest Statement

The authors declare that the research was conducted in the absence of any commercial or financial relationships that could be construed as a potential conflict of interest.
